# Nutrition status of children in Latin America

**DOI:** 10.1111/obr.12571

**Published:** 2017-07-25

**Authors:** C. Corvalán, M. L. Garmendia, J. Jones‐Smith, C. K. Lutter, J. J. Miranda, L. S. Pedraza, B. M. Popkin, M. Ramirez‐Zea, D. Salvo, A. D. Stein

**Affiliations:** ^1^ Institute of Nutrition and Food Technology University of Chile Santiago Chile; ^2^ Department of Health Services, School of Public Health University of Washington Seattle WA USA; ^3^ Pan American Health Organization USA; ^4^ CRONICAS Center of Excellence in Chronic Diseases Universidad Peruana Cayetano Heredia Lima Peru; ^5^ Center for Nutrition and Health Research National Institute of Public Health Cuernavaca Mexico; ^6^ Carolina Population Center The University of North Carolina at Chapel Hill Chapel Hill NC USA; ^7^ INCAP Research Center for the Prevention of Chronic Diseases (CIIPEC) Institute of Nutrition of Central America and Panama Guatemala; ^8^ Michael and Susan Dell Center for Healthy Living The University of Texas Health Science Center at Houston, School of Public Health, Austin Campus Austin TX USA; ^9^ Hubert Department of Global Health of the Rollins School of Public Health Emory University Atlanta GA USA

**Keywords:** childhood obesity, children, Latin America, nutrition and physical activity situation

## Abstract

The prevalence of overweight and obesity is rapidly increasing among Latin American children, posing challenges for current healthcare systems and increasing the risk for a wide range of diseases. To understand the factors contributing to childhood obesity in Latin America, this paper reviews the current nutrition status and physical activity situation, the disparities between and within countries and the potential challenges for ensuring adequate nutrition and physical activity. Across the region, children face a dual burden of undernutrition and excess weight. While efforts to address undernutrition have made marked improvements, childhood obesity is on the rise as a result of diets that favour energy‐dense, nutrient‐poor foods and the adoption of a sedentary lifestyle. Over the last decade, changes in socioeconomic conditions, urbanization, retail foods and public transportation have all contributed to childhood obesity in the region. Additional research and research capacity are needed to address this growing epidemic, particularly with respect to designing, implementing and evaluating the impact of evidence‐based obesity prevention interventions.

## Introduction

Currently, more than 20% (approximately 42.5 million) of Latin American children aged 0 to 19 years are overweight or obese 1. Without substantial changes, this rate will only continue to rise unabated. Along with the growth in overweight and obesity comes an increase in concomitant non‐communicable diseases such as diabetes, cardiovascular disease and some forms of cancer 2. As a result, the disabilities and mortality associated with non‐communicable diseases will start to occur earlier in adulthood 3.

This epidemic is the result of a number of socioeconomic, cultural and demographic changes that have occurred in Latin America in the last decades. Improvements in socioeconomic conditions, increases in women's employment 4, rapid urbanization 5, the proliferation of the food retail and food services sector and increasing use of private transportation have interacted in complex ways to influence diet and physical activity patterns and ultimately the nutrition status of children in the region. The aim of the present article is to describe the current nutrition, dietary and physical activity status of children in the region considering disparities within and between countries and barriers and facilitators that may impact present and future efforts to ensure adequate nutrition and prevent childhood overweight and obesity. This is the first of a series of papers derived from the NIH sponsored workshop: ‘Preventing Childhood Overweight and Obesity in Latin America: Linking Evidence to Policy and Practice’.

## Nutrition status of children in the Latin American region

The current nutrition situation of the region reflects the fact that most of the recent economic, cultural and demographic changes 6, 7 have not impacted the population equally, resulting in a scenario where undernutrition (primarily stunting) exists alongside overweight and obesity, with micronutrient malnutrition across both conditions. In Latin America, stunting (height‐for‐age below −2 standard deviation [SD]) is the most common nutritional deficit in children aged 0–59 months 8, although it varies widely between countries with a prevalence as high as 48% in Guatemala and as low as 1.8% in Chile. At the same time, overweight (weight‐for‐height over +2 SD) is also widespread among children under 5 years old, particularly in the Southern Cone and in Mexico (Table 1) 1, 9. For older children, there is less systematic evidence, but available reports and articles mainly in adolescent girls suggest that the prevalence of overweight and obesity exceed 25% of the population in some countries 1, 10, 11 (Table 1). The coexistence of high prevalence of stunting and high prevalence of overweight at the national level also increases the likelihood of observing double burden at the household level, such as stunted children with overweight/obese mother (Table 2) 8. Moreover, both conditions can be present at the individual level; stunted and overweight children are observed although at low levels (Table 2) 11, 12.

**Table 1:** Prevalence of stunting and overweight in children in Latin America 1

Country	Children <5 years			Children >5 years			
	Year	Stunting (%)	Overweight (%)	Year	Stunting (%)	Year	Overweight (%)
Argentina	2005	8.2	9.9	–	–	–	–
Barbados	2012	7.7	12.2	–	–	–	–
Belize	2006	22.2	13.7	–	–	–	–
Bolivia	2008	27.2	8.7	2003	24.7	–	–
Brazil	2007	7.1	7.3	–	–	2008–09	20.5
Chile	2014	1.8	9.3	–	–	2005	31.0
Colombia	2010	12.7	4.8	2005	11.2	2010	16.7
Costa Rica	2008	5.6	8.1	–	–	–	–
Cuba	2000	7.0	–	–	–	–	–
Dominican Republic	2013	7.1	7.6	–	–	–	–
Ecuador	2012	25.2	7.5	2012	19.1	2012	26.0
El Salvador	2014	14.0	6.0	2003	28.7	–	–
Guatemala	2009	48.0	4.9	–	–	–	–
Guyana	2014	12.0	5.3	–	–	–	–
Haiti	2012	21.9	3.6	2005	6.5	–	–
Honduras	2012	22.7	5.2	2005	25.0	–	–
Jamaica	2012	5.7	7.8	–	–	–	–
Mexico	2012	13.6	9.0	–	–	2012	43.9
Nicaragua	2006	23.0	6.2	2001	19.7	–	–
Panama	2008	19.1		–	–	–	–
Paraguay	2012	10.9	11.7	–	–	–	–
Peru	2013	18.4	7.2	2004–2008	32.1	–	–
Suriname	2010	8.8	4.0	–	–	–	–
Trinidad and Tobago	2000	5.3	4.9	–	–	–	–
Uruguay	2011	10.7	7.2	–	–	–	–

Stunting: height‐for‐age *z* score <2, WHO 2007.

Overweight: weight‐for‐height *z* score >2, WHO 2007.

**Table 2:** Prevalence of dual burden of malnutrition in the Latin America region 8

Country	Year	Household level (Stunted child <5 years^1^ and mother excess weight^2^)	Individual level children prevalence of double burden (%)		
		Prevalence of double burden (%)	Age range (years)	Type of double burden	Prevalence of double burden (%)
Brazil	2006–07	2.7	5–11	Overweight^3^	1.0
Colombia	2010	5.1	5–12	Overweight^4^	0.1
Ecuador	2012	13.1	5–11	Overweight^4^	2.8
Guatemala	2008	20.0 (overall)	<5	Overweight^5^	1.2 (overall)
		28.2 (indigenous)			2.8 (indigenous)
México	2012	8.4	5–11	Overweight^3^	1.0
Uruguay	2004	6.3	6	Overweight^4^	1.9

1 Height‐for‐age *z* score <2.

2 Body mass index ≥25 kg/m^2^.

3 Body mass index‐for‐age >1 *z* scores.

4 Body mass index‐for‐age >2 *z* scores.

5 Weight‐for‐height >2 *z* scores.

Within countries, nutritional disparities vary with ethnicity, the degree of urbanization 13, 14 and socioeconomic status (Table 3) 15, 16. Nearly 13% of the entire Latin American population and approximately 40% of the rural population are indigenous 17. However, the impact of ethnicity in the growing obesity epidemic has not been well characterized. Studies have reported differences in nutritional studies by ethnicity in Guatemala 18, Chile 19, 20 and Mexico 21, among other Latin American countries, but the direction and magnitude of the associations vary across studies. Urban–rural differences on overweight and obesity prevalence have also been described using representative data of children under 5 years of age (Table 3). The data show that in some countries in the region, such as Honduras and Peru, urban areas experience a higher prevalence of overweight and obesity compared with rural areas; similar results have been described for girls 10–19 years old 10. However, in other countries, such as Bolivia and Nicaragua, the opposite is true; with boys from rural areas having the highest prevalence rates. The prevalence of overweight and obesity also differs by maternal education. In most countries, preschool children with mothers with higher education have higher prevalence of overweight and obesity (Table 3); however, there are recent reports that suggest a shift to the poor at least in adults from low‐middle income countries with higher economic growth 15, 22, 23. Overall, these results highlight the complexity and the diversity of each country's local context and thus, the need of better understanding these realities when defining evidence‐based interventions to improve nutrition in the region 24, 25.

**Table 3:** Prevalence of overweight^a^ among children age 0–5 years according to gender and by urbanicity and by socioeconomic status

	Bolivia (2008)		Colombia (2010)		Dominican Republic (2013)		Honduras (2011)		Nicaragua (2001)		Peru (2012)	
	Boys	Girls	Boys	Girls	Boys	Girls	Boys	Girls	Boys	Girls	Boys	Girls
	(*n* = 3939)	(*n* = 3939)	(*n* = 8119)	(*n* = 7,693)	(*n* = 1621)	(*n* = 1554)	(*n* = 5152)	(*n* = 4774)	(*n* = 3009)	(*n* = 2902)	(*n* = 4637)	(*n* = 4527)
Overall, % (*n*)^b^	12.4 (472)	10.2 (371)	5.7 (457)	4.9 (355)	9.5 (142)	8.1 (118)	6.1 (298)	5.3 (235)	9.5 (315)	7.4 (239)	9.5 (253)	7.0 (253)
Rural, % (*n*)	13.4 (250)	10.8 (189)	5.2 (152)	4.2 (121)	9.2 (39)	7.7 (32)	5.2 (176)	4.1 (103)	10.4 (184)	7.8 (144)	6.2 (116)	4.0 (65)
Urban, % (*n*)	11.4 (222)	9.6 (182)	5.9 (305)	5.1 (234)	9.6 (103)	8.2 (86)	7.1 (122)	6.8 (132)	8.2 (131)	7.1 (95)	11.2 (270)	8.6 (188)
Maternal education												
Less than primary school, % (*n*)	13.2 (32)	7.1 (15)	3.0 (7)	3.5 (7)	14.4 (3)	1.2 (1)	3.9 (14)	3.8 (12)	10.7 (85)	7.0 (58)	5.9 (7)	2.9 (6)
Primary school, % (*n*)	13.1 (258)	10.0 (204)	3.9 (105)	3.6 (80)	8.7 (35)	5.2 (24)	5.3 (163)	4.2 (121)	8.7 (127)	7.6 (107)	7.2 (99)	4.3 (47)
Secondary school, % (*n*)	11.0 (117)	10.8 (104)	6.2 (258)	5.3 (204)	8.82 (58)	8.2 (47)	6.6 (98)	6.7 (81)	8.7 (79)	7.5 (60)	7.8 (155)	8.3 (131)
Higher than secondary school complete, % (*n*)	11.8 (65)	11.4 (48)	7.0 (87)	5.6 (64)	11.2 (46)	11.9 (46)	14.9 (23)	12.0 (21)	14.8 (24)	7.4 (14)	16.2 (125)	8.2 (69)

a Percentage of children overweight for their age (above +2 SD of weight for height according to the WHO standard).

b Proportion of excess weight is estimated using sampling weights; unweighted *N* is provided in parentheses.

Source: Authors' analysis of Demographic Health Surveys.

## The nutrition transition

### Dietary changes

One of the main drivers of the current obesity epidemic among children in Latin America is the rapid dietary change over the last few decades, commonly referred to as the nutrition transition 26. Information on children's dietary changes is limited; however, based on the relationship between parent and child food consumption habits, we are able to extrapolate from adult data findings 27. Across Latin America, intake of energy, fat and protein has increased substantially over the last few decades 28, 29, 30, 31. For example, in Guatemala, Chile, Mexico and Brazil, the consumption of animal products and sugar‐sweetened beverages (SSB) has increased, while the intake of nutrient‐rich vegetables and legumes has decreased. The result of these changes is a diet high in fat and sugar but low in micronutrients like vitamin A and zinc, which is associated with increased risk for infection and compromised immune system 32.

**Figure 1:**
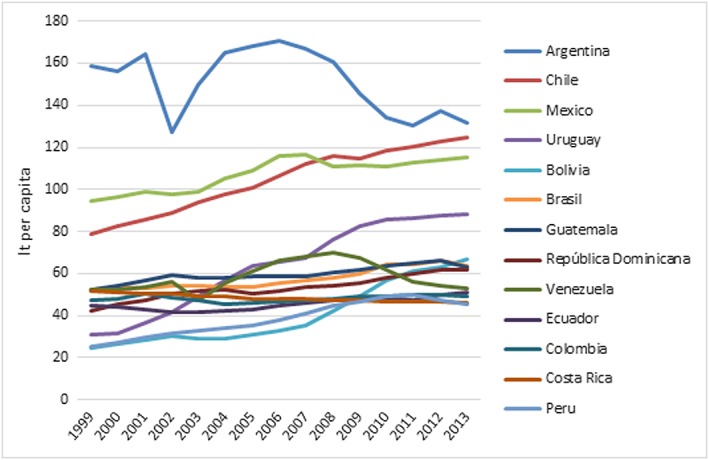
Trends in sales of processed beverages in Latin American countries, Euromonitor 1999–2013. Source: Euromonitor International. [Colour figure can be viewed at wileyonlinelibrary.com]

**Figure 2:**
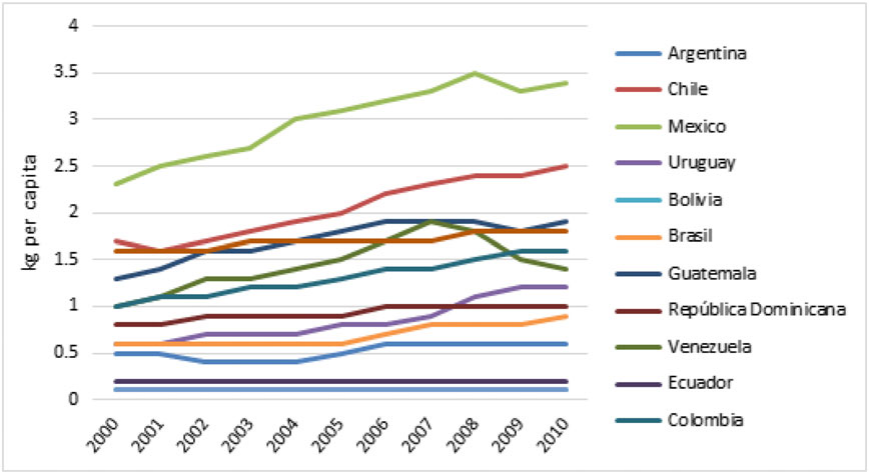
Trends in sales of processed foods in Latin American countries, Euromonitor 2000–2010. Source: Euromonitor International. [Colour figure can be viewed at wileyonlinelibrary.com]

Without substantial intervention, sales of SSB and processed foods are only expected to continue rising across most countries in the region (Figs. 1 and 2) 33, 34. Three of the world's top five SSB consuming nations are in Latin America, and the region as a whole is experiencing increasing levels of SSB intake 34. SSB have been linked to increased childhood obesity risk 35, 36, 37. Recent data from Mexico show that for children aged 1 to 19 years, SSB represent 17.5% of their total daily energy intake; an increase of at least 45.3 Kcal per day, which is equivalent to an excess 2.5 pounds per month, between 1999 and 2012 38, 39, 40. Similarly, sales of energy‐dense, nutrient‐poor foods such as snacks, SSB and frozen ready‐to‐heat foods have steadily increased since 1998 in many countries in the region 41. In Brazil, these foods have become an increasingly important part of the food expenditure 42, 43 that rose approximately 40% between 1990 and 2010 and now constitute approximately 25% of total calorie intake 41. Similarly, in Chile, increased food expenditure in SSB, ready‐to‐eat meals and eating outside of the home has increased over the last years (Fig. 2) 43. In Guatemala, a 10 percentage point increase in the share of household expenditures on these food products was associated with an increase in body mass index (BMI) of family members 10 years old and older by 4.25%, suggesting that consumption of these foods is a major risk factor for obesity. Therefore, halting consumption of these foods should be a target for intervention strategies 44.

### Changes in food environment

Dietary changes observed in Latin America are the result of a series of changes in the global, national and local food environments that affect availability, accessibility and affordability of foods, as well as people's food demands through higher exposure to advertisement and marketing 45.

#### Global food environment

Several studies have examined the association between economic globalization and the types of foods and food outlets available in local environments. Trade liberalizations between the USA and Latin America since the 1990s have increased the ease with which multinational companies can export foods 46, 47. Compared to the 30 years prior to the North American Free Trade Agreement, the amount of corn, meats (beef/veal, chicken and pork), soybeans, sugars and snack foods exported from the USA to Mexico has increased importantly 48. Trade liberalization is hypothesized to have played a key role in the expansion of supermarkets throughout Latin America 49. Better understanding of how trade and investment agreements impact diets in the region may result critical for ensuring healthy food environments and improving population's nutrition.

#### National food environment

Supermarkets tend to provide more packaged, ready‐to‐eat or ready‐to‐heat foods manufactured by multinational food corporations as opposed to traditional open‐air markets. In‐store audits have revealed that the foods occupying most of the space in these stores were cookies, SSB, potato chips and canned foods; these foods also tended to be prominently displayed 50. This change in the retail sales of food has been a major driver in shifting the diets of Latin American populations 51. Between 1990 and 2000, supermarkets in Latin America went from occupying 10–20% of the retail share to 50–60%. In most Latin American countries, multinational supermarkets now comprise 60–80% of the top five chains in each country 49 and their growth continues, particularly with their penetration into villages 52. For example, one group looked at the change in retail and subsistence food environments over 15 years in Maycoba, a rural town in Sonora, Mexico, with approximately 1,100 people, many of whom are of indigenous Pima Indian descent 53. Results found that the number of small grocery stores increased from 6 to 11. Moreover, the presence of multinational fast‐food chains has grown exponentially in Latin America. In 1995, the region had 100 McDonald's; by 2014, this had increased to 2,268 54, 55. Fast‐food consumption has been linked to higher energy consumption and poorer diet quality in US children and adolescents 56.

#### Neighbourhood food environment

It is believed that community food environments influence dietary behaviours 57; however, the exact mechanisms and strength of the associations remain controversial given the difficulty in standardizing study designs, data collection methods and statistical analyses 58. In Guatemala 59, shopping at a supermarket is associated with increased consumption of pastries, chocolates and ice cream and decreased consumption of corn and beans, particularly in poor households 59. These results are in line with studies carried out in the USA 60, 61 and other developed countries 62. However, in developing countries, evidence of the net effect of the local food retail environment on people's nutritional status remains controversial 63, 64, 65. In the case of Latin America, developing adequate measurement tools to assess the retail food environment requires the consideration of open‐air markets 66 as they are highly prevalent in the region 67.

#### School food environment

In Brazil, public schools have higher proximity to stores that sell energy‐dense, nutrient‐poor products than natural foods 68. With some exceptions, studies indicate that higher proximity and density of fast‐food and grocery stores to schools are linked to more fast‐food purchases 69 and poorer‐dietary scores in adolescents 70. However, this finding is not consistent across the research landscape 71, 72. In addition, street‐food vending is a common practice in Latin America 73. Recently, a study conducted in 60 schools in two of Mexico's major cities showed that there were on average six mobile food vendors around public schools and that the number of mobile food vendors around schools was positively associated with the children's BMI 74.

There is also strong evidence that the in‐school environment shapes diets and therefore, the nutritional status among children 75, 76. Recently, a representative sample of Brazilian adolescents showed that availability of energy‐dense, nutrient‐poor foods, such as SSB and salty snacks inside the schools was associated with higher consumption of these food products 77. In Mexico, it has been also described that the number of stores inside of schools is positively associated with higher BMIs among school children 74.

#### Food marketing exposure to children

Advertising has been shown to strongly influence consumer choices and likely plays a role in changing diets in Latin America 78, 79, 80. It has been previously reported that in Mexico, where approximately 95% of homes have a television, the food group most frequently advertised is SSB (24.6%), followed by chocolate and confectionery sugar (19.7%), cakes, sweet biscuits and pastries (12.0%), savoury snacks (9.3%), breakfast cereals (7.1%), ready‐to‐eat food (6.4%) and dairy products (6.0%) 81. In addition to television advertising, food companies use the licenced television characters on their packaging to promote their products to children. In Guatemala, a study shows that children prefer the taste of food from packages with a licenced character compared with the same food in packages without the character 82. In several countries, advertising energy‐dense, nutrient‐poor food products is not only allowed but also highly prevalent in stores within walking distance of schools and kiosks inside schools 83, 84, 85.

## Physical activity changes

Physical activity levels have decreased worldwide likely because of the adoption of a sedentary lifestyle 86. A number of physical activity factors have contributed to the global epidemic of obesity, namely, (i) reduced walking for transportation as a result of improved access to motorized transportation; (ii) decreased occupational physical activity due to automated processes, leading to more sedentary occupation patterns; and (iii) increased time spent in sedentary leisure activities 45, 46, 47, 50, 86, 87, 88, 89, 90, 91. It is difficult to describe children's physical activity status because few countries have established surveillance systems to assess population‐level, physical activity using representative samples and consistent measures, and the ones that do are targeted to adults 92, 93.

### Physical inactivity and sedentary behaviour in Latin America

Given the cost associated with using objective measures like accelerometers and the lack of research in this field in most Latin American countries 94, the prevalence of physical inactivity among children in the region remains not well known. However, findings pertaining to adults and older adolescents indicate that Latin America is the most inactive region in the world with 43% of those over 15 years old categorized as inactive 92. In almost all countries of the region, at least a third of the population over 15 years old is physically inactive except in Guatemala (16.2%) and Dominica (24.4%) 92. Recently, the Active Healthy Kids Canada Report Card on Physical Activity for Children and Youth was replicated in 14 countries, including Colombia and Mexico 95. In Mexico, it was reported that 41% of children and young adolescents 10 to 14 years old participated in at least one organized sport during the previous year and 66% walked to school 96. In Colombia, only 6.1% of preschool and school‐aged children, 3 to 12 years old, received physical education classes from a professional instructor 97. Television viewing has been commonly used as a proxy measure for sedentary behaviour among children 98. Studies in Latin America suggest that the mean television watching time is 3 to 4 h per day, with the majority of children in the region spending more than 2 h per day in front of the television. In almost all countries, there is a significant association between television viewing time and childhood obesity 99, 100, 101.

### Changes in physical activity environment

#### National physical activity environment

Latin America is the most urbanized region in the world with almost 80% of Latin Americans living in urban areas 5. Over the past decades, the region has transitioned from the more generalized use of public transit systems to privately owned motorized vehicles 102. In most Latin American cities, motor vehicle ownership remains low compared with high‐income countries (HIC), but this is rapidly changing. Findings from a global, 12‐country study report found that the Latin American region has the lowest levels of physical activity as a form of leisure and was among the highest to use physical activity as a form of transport 94. Because activity in the region seems to be necessity‐driven vs. choice‐driven, increases in motor vehicle ownership among Latin Americans is of concern in terms of its effect on activity levels. Among children, active travel to school is still relatively high in some countries, such as Mexico 103; however, this is changing rapidly as a result of increased motor vehicle ownership and growing safety‐related concerns in the region 104.

Additionally, technological trends are influencing physical activity behaviours 91, 105, 106, 107. Increasingly, new technologies that encourage sedentary leisure, like tablets, smartphones and internet‐connected video game systems, are becoming ubiquitous. Latin America has seen a strong increase in technology sales in the last few years 108. However, the widespread availability of these technologies in Latin America also makes them a potential vehicle to deliver interventions to promote physical activity and healthy diets, particularly among adolescents 109. A recent study demonstrated that while one mobile health technology with advice for lifestyle improvements did not reduce blood pressure, it successfully promoted a small reduction of weight and improved some dietary habits in individuals with prehypertension living in low‐resource urban settings in Argentina, Peru and Guatemala 110. Although evidence is currently limited, the recent popularity of interactive games that encourage physical activity, like Pokémon Go, could present opportunities to use technology to improve health; although sustaining effects in the long term may be an issue 111.

#### Neighbourhood physical activity environment

There is significant evidence from HIC linking physical activity with the built and social environments but relatively few studies from Latin America particularly among children 93, 94, 112. The findings from HIC suggest that the relationship between physical activity and the built environment may be context specific. For instance, evidence from these countries shows positive associations between mixed land‐use, residential density and street connectivity (i.e. US‐based walkability index), and physical activity 113. Yet in Latin America and other low‐ and middle‐income countries, findings have been inconclusive 94, 114, 115. Studies from Brazil, Colombia, and Mexico, have identified key environmental constructs that relate to activity levels among Latin Americans, such as aesthetics, public spaces for activity, social cohesion and safety 116, 117. Some very successful and innovative programmes to promote physical activity have emerged from Latin America, such as *Ciclovias*, the open‐street programmes, in Colombia 118 and *Academia da Saude*, a national programme that offers physical activity classes, nutrition counselling, among other activities 119. These programmes have been extensively evaluated, and findings show that they have a positive impact on the activity levels of children, adults and seniors 118, 120, 121, 122, 123.

#### School physical activity environment

Evidence of the impact of school environment on physical activity levels is also scarce in Latin America and other low‐ and middle‐income countries. However, from evidence of the USA, we know that most of vigorous physical activity as well as sedentary behaviours are concentrated in the school environment 124, 125. The ease of walking and biking on a campus has been related to US college students' walking behaviour and BMI 126; Higher vigorous physical activity in the schools is associated with higher daily vigorous activity suggesting that increasing school‐based physical activity could be an effective intervention for increasing overall physical activity in youth 127. A recent systematic review has shown that school‐based interventions that are based on mandatory physical activity are the ones more likely to increase moderate to vigorous physical activity in youth (i.e. 23 min per day that corresponds to almost 40% of the WHO physical activity recommendation for this age group), although other less intensive actions also result in significant increases of moderate to vigorous physical activity: classroom activity breaks (19 min); afterschool activity programmes (10 min); standardized physical education curricula (6 min more than traditional physical education); modified playgrounds (6 min); and modified recess (5 min more than traditional recess) 128. A review that included information of five school‐based physical education programmes from Latin America (Brazil, Chile and US/Mexico border) shows that different types of interventions have the potential of increasing physical activity levels during physical activity classes and active transportation to schools 129.

## Challenges of addressing the childhood obesity epidemic

Paediatric healthcare systems are stressed by this new nutritional scenario, where overweight as well as its associated consequences are increasingly observed at progressively younger ages 130. Healthcare systems – from both the human and infrastructure perspectives – need to rapidly adapt to prevent, identify and treat new conditions associated with overweight and obesity 131 forcing already frail public health structures to respond to complicated childhood health concerns. Existing monitoring systems, traditionally focused on monitoring weight as a way of tracking underweight (weight‐for‐age below −2 SD), must adapt to incorporate length and height measurements if excess weight is to be adequately monitored. Failure to incorporate length to monitoring systems may result in unintendedly missing the increasing rates of overweight, which is a measure of weight‐for‐height 132.

In Latin America, social programmes, such as food and nutrition and cash transfer programmes, have played an important role in addressing poverty and decreasing undernutrition; however, there is a concern that these programmes may need to be adapted to avoid inadvertently contributing to an increase in the risk of becoming overweight 133, 134, 135. The presence of the dual burden of nutrition requires designing policies and programmes to address nutrition status at both ends of the spectrum – from undernutrition to overweight – at the individual, household and societal levels 24, 136. Presently, in some countries, there are efforts to address the growing obesity epidemic by modifying social and nutrition programmes or improving public school environments in general 137, 138. However, in other countries such as Guatemala and Peru, social programmes are still mainly oriented to fighting undernutrition 138, 139.

Culturally appropriated, local evidence that considers the particularities of the food and physical activity environment of the region is needed to define effective preventive actions to address the growing obesity epidemic. Unique subpopulation consideration must also be included, especially in Latin America, where nearly 13% of the entire Latin American population is indigenous, a population with known nutritional differences 15, 16, 17, 19, 20. Presently, several large‐scale actions such as the taxation of SSB, use of front‐of‐package warning messages, marketing regulations and so on are taking place in the region 118, 140, 141, 142, 143, 144; however, rigorous evaluations are needed to assess their impact and guide adjustments or additions to maximize their effectiveness.

## Conclusion

In Latin America, there is a coexistence of undernutrition and overweight among children that importantly differ between and within countries. Rapid urbanization combined with greater penetration of the retail food and food service sector has promoted diets that rely on energy‐dense, nutrient‐poor foods. At the same time, sedentary behaviours have become the norm among children. If no action is taken, the rates of overweight and obese children will continue to rise. As explored in subsequent articles in this supplement, a new research agenda accompanied by strengthened translation of the research into policy and practice and increased research capacity are needed in the region to confront this growing epidemic.

## Conflict of interest statement

The Fogarty International Center at the US National Institutes of Health sponsored travel for each, non‐local author to attend the ‘Preventing Childhood Overweight and Obesity in Latin America: Linking Evidence to Policy and Practice’ workshop, the precursor for this article. The following authors declare a further conflict of interest as specified in their ICMJE disclosure: Camila Corvalán, Chessa K Lutter, Barry M. Popkin, Manuel Ramirez‐Zea, Deborah Salvo and Aryeh D Stein.
